# Hydrostatic pressure effect on magnetic phase transition and magnetocaloric effect of metamagnetic TmZn compound

**DOI:** 10.1038/srep42908

**Published:** 2017-02-16

**Authors:** Lingwei Li, Guanghui Hu, Yang Qi, Izuru Umehara

**Affiliations:** 1 Key Laboratory of Electromagnetic Processing of Materials (Ministry of Education), Northeastern University, Shenyang 110819, China; 2 Institute of Materials Physics and Chemistry, School of Materials Science and Engineering, Northeastern University, Shenyang 110819, China; 3 Department of Physics, Faculty of Enginnering, Yokohama National University, Yokohama 240-8501, Japan

## Abstract

The magnetocaloric effect (MCE) is an intrinsic thermal response of all magnetic solids which has a direct and strong correlation with the corresponding magnetic phase transition. It has been well recognized that the magnetic phase transition can be tuned by adjusting applied pressure. Therefore, we perform the high hydrostatic pressure magnetization measurements (up to 1.4 GPa) on a recently reported giant MCE material of TmZn. The results indicate that the Curie temperature of *T*_C_ increases from 8.4 K at the ambient pressure to 11.5 K under the pressure of 1.4 GPa. The field-induced first order metamagnetic transition is getting weak with increasing pressure, which results in a reduction of MCE. The hydrostatic pressure effect on the magnetic phase transition and MCE in the metamagnetic TmZn is discussed.

In recent years, the magnetocaloric effect (MCE) in magnetic materials has been well investigated, not only due to their potential applications for active magnetic refrigeration but also enable to understand the related fundamental properties of these materials^1^,^2^,^3^,^4^,^5^,^6^,^7^,^8^. MCE is an intrinsic thermal response of all magnetic solids which manifests as the isothermal magnetic entropy change (Δ*S*_M_) and the adiabatic temperature change (Δ*T*_ad_) when the magnetic field is applied or removed. Magnetic refrigeration technology based on MCE is an alternative technology over the commercial gas compression/expansion refrigeration because of its promising advantages (high energy efficiency, environmental conservation, small noise, *etc*.)^1^,^2^,^3^,^4^.

The MCE is the essential result of the magnetic entropy change due to the coupling of a magnetic spin system with magnetic field, and it is significant around the magnetic phase transition. Despite it has been well recognized that the magnetic phase transitions can be tuned by pressure^9^,^10^,^11^, only a few works are related to the hydrostatic pressure effect on MCE^12^,^13^,^14^,^15^,^16^,^17^. Morellon *et al*^12^. found that the external pressure can tune the magnetic phase transition and induce a giant MCE in Tb_5_Si_2_Ge_2_, whereas the MCE in Gd_5_Ge_2_Si_2_ decreases evidently with increasing pressure^13^. The peak position of the magnetic entropy change Δ*S*_M_ for La_0.69_Ca_0.31_MnO_3_ shifts to higher temperatures gradually, while the maximum value of −Δ*S*_M_ is almost unchanged with increasing pressure^14^. As a matter of fact, a weak pressure dependence on MCE has also been reported in some MCE materials, such as, GdCo_2_B_2_^15^ and GdCr_2_Si_2_^16^ compounds. Very recently, a giant reversible MCE in metamagnetic TmZn compound was reported^18^. To further understand the magnetic phase transition and its correlation with MCE, in this paper, we have further performed the high hydrostatic pressure magnetization measurements on TmZn.

## Results and Discussion

**Figure 1:**
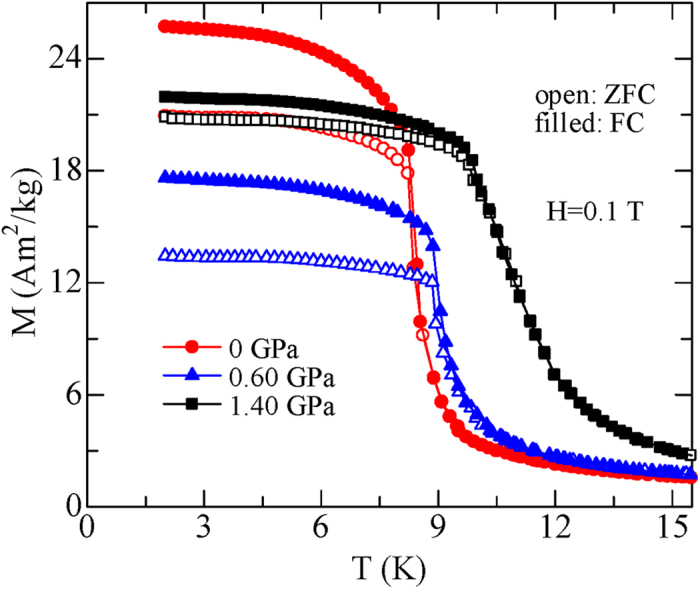
Temperature dependence of the zero field cooled (ZFC) and field cooled (FC) magnetization (*M*) under a magnetic field of 0.1 T for TmZn under the pressures of 0, 0.60 and 1.40 GPa.

Figure 1 shows the temperature dependence of the zero field cooled (ZFC) and field cooled (FC) magnetization (*M*) measured in a magnetic field (*H*) of 0.1 T for TmZn under various hydrostatic pressures. All the ZFC and FC *M*(*T*) curves show a paramagnetic to ferromagnetic (PM-FM) transition. The values of the Curie temperature *T*_C_ (defined as the minimum of d*M*/d*T* vs. *T*) are determined to be 8.4, 9.1 and 11.2 K under the pressures of 0, 0.60 and 1.40 GPa, respectively. The value of *T*_C_ in zero pressure is well consistent with previous reported results^18^,^19^. The magnetic properties of TmZn have been extensively investigated thirty years ago by the specific heat, resistivity, magnetization and neutron diffraction measurements^18^,^19^,^20^,^21^,^22^. The results indicated that the strong field and temperature dependence of magnetic moment in TmZn cannot be described by a simple Rdderman-Kittel-Kasuya-Yosida (RKKY) model, and the low temperature ferromagnetic state in TmZn is probably due to the soft longitudinal spin fluctuations^23^, since the low temperature magnetization is not saturate even under fields approaching 10 T^18^.

**Figure 2:**
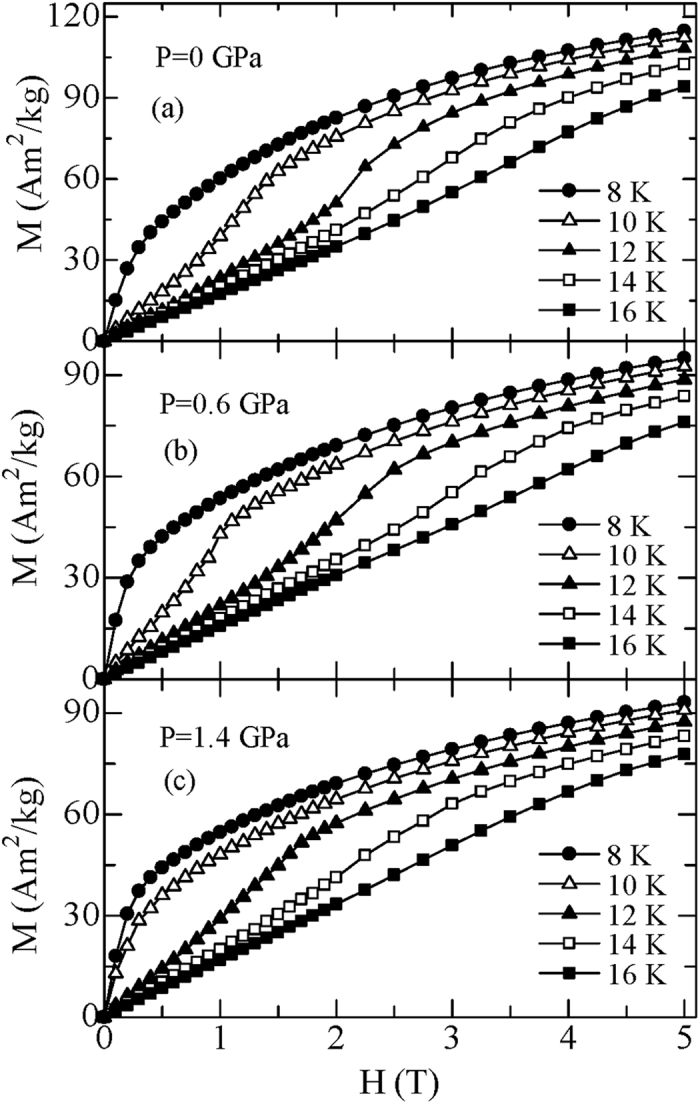
Magnetic field dependence of the magnetization *M*(*H*) curves for TmZn at some selected temperatures under the pressures of (**a**) 0, (**b**) 0.60 and (**c**) 1.40 GPa, respectively.

**Figure 3:**
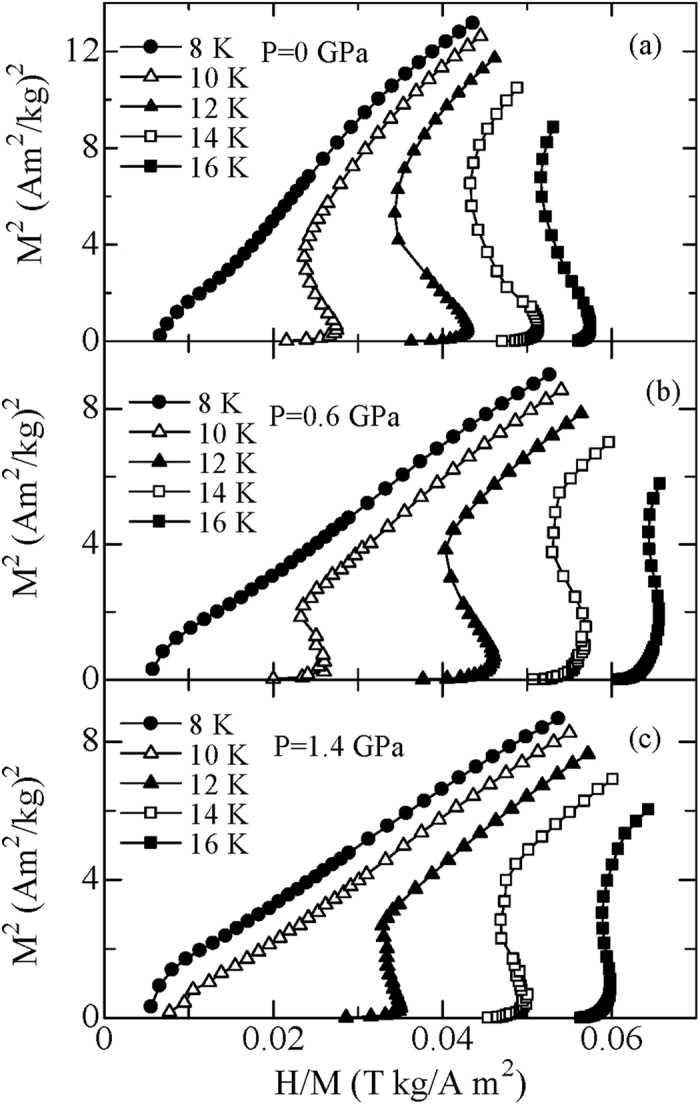
The Arrott plots (*H*/*M* versus *M*^2^) for TmZn at some selected temperatures under the pressures of (**a**) 0, (**b**) 0.60 and (**c**) 1.40 GPa, respectively.

To investigate the pressure effect on MCE in TmZn, a set of magnetic isothermal *M*(*H*) curves under the hydrostatic pressures of 0, 0.60 and 1.40 GPa with increasing and decreasing magnetic field up to 5 T for TmZn are measured. No obvious hysteresis can be observed under all the pressures for the whole temperature range. To ensure the readability of the figure, only several selected isotherms with increasing field for TmZn under 0, 0.60 and 1.40 GPa are presented in Fig. 2 for a comparison, and the corresponding Arrott plot (*H*/*M* versus *M*^2^) curves are given in Fig. 3. Except some differences in values, the magnetic isotherms and the Arrott plots show a similar behavior under all the present pressures. I. e., a field-induced metamagnetic transition appears in a certain temperature range (around and above *T*_C_), and the critical field shifts to higher magnetic fields with increasing temperature. Based on the Banerjee criterion^24^, the magnetic transition is first order if some of the *H*/*M* versus *M*^2^ curves show negative slope at some points. Therefore, the present TmZn under all the present pressures reveal a typical field-induced first order metamagnetic transition, since a clear S-shape can be observed in the Arrott plots under all the pressures (as given in Fig. 3). In details, the magnetization jump during the metamagnetic transition and the temperature range of the metamagnetic transition is getting smaller with increasing pressure. Additionally, the slop of the Arrott plot related to the strength of first order transition is getting weak with increasing pressure. These behaviors indicate that the first order metamagnetic transition of TmZn is suppressed gradually with increasing hydrostatic pressure but not breakdown up to 1.40 GPa.

**Figure 4:**
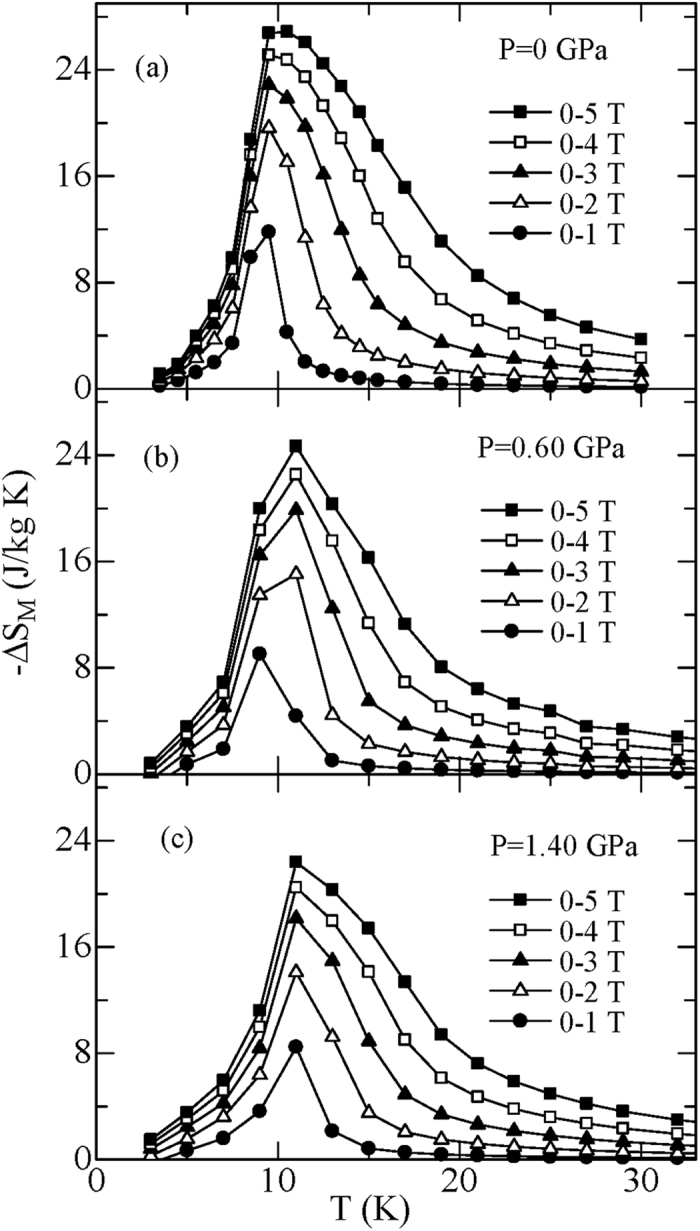
Temperature dependence of magnetic entropy change −Δ*S*_M_ for TmZn under the pressures of (**a**) 0, (**b**) 0.60 and (**c**) 1.40 GPa, respectively.

Figure 4 presents the magnetic entropy change Δ*S*_M_ for TmZn under various pressures which is calculated by integrating the Maxwell’s relation, 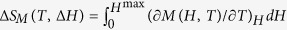, using the data of magnetization isotherms *M (H, T*). As expected, −Δ*S*_M_ exhibits a pronounced peak around *T*_C_ where the magnetization changes rapidly with varying temperature; and the peak position of −Δ*S*_M_ shifts to higher temperatures gradually which is a consequence of pressure induced *T*_C_ shifts. The values of maximum magnetic entropy change (−Δ*S*_M_^max^) for TmZn under the pressures of 0, 0.60 and 1.40 GPa are evaluated to be 11.8, 9.1 and 8.5 J/kg K for the field change of 0–1 T, to be 19.6, 15.1 and 14.1 J/kg K for the field change of 0–2 T, and to be 26.9, 24.7 and 22.4 J/kg K for the field change of 0–5 T, respectively. I. e., the MCE decreases gradually with increasing pressure. Apparently, the temperature dependence of −Δ*S*_M_ for TmZn is getting flatter and more symmetrical with increasing pressure, this is another signal of the first order magnetic phase transition is getting weak with increasing hydrostatic pressure. It is well known that the MCE has a direct and strong correlation with the corresponding magnetic phase transition. Therefore, the reduction MCE in present TmZn is related to the suppression of the first order metamagnetic transition by the applied hydrostatic pressure. Another important parameter for MCE materials is the refrigerant capacity (*RC*) which can be evaluated by numerically integrating the area under the −Δ*S*_M_ (T) curve at half maximum of the peak taken as the integration limits, 
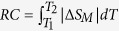
, where *T*_1_ and *T*_2_ are the temperatures of the cold end and the hot end of an ideal thermodynamic cycle, respectively^4^. For the field change of 0–5 T, the values of *RC* for TmZn are 214, 203 and 141 J/kg under the pressures of 0, 0.60 and 1.40 GPa, respectively.

## Conclusions

In summary, the magnetic phase transition and magnetocaloric effect in metamagnetic TmZn have been systematically investigated by magnetization measurements under high hydrostatic pressure up to 1.4 GPa. The Curie temperatures of *T*_C_ are determined to be 8.4, 9.1 and 11.2 K under the pressures of 0, 0.60 and 1.40 GPa, respectively. The field-induced first order metamagnetic transition in TmZn is suppressed gradually with increasing hydrostatic pressure but not breakdown up to 1.40 GPa. The MCE in TmZn decreases gradually with increasing pressure. For a magnetic field change of 0–5 T, the maximum values of the magnetic entropy change of TmZn are determined to be 26.9, 24.7 and 22.2 J/kg K under the pressures of 0, 0.60 and 1.40 GPa, respectively. The corresponding values of *RC* are evaluated to be 214, 203 and 141 J/kg.

## Methods

The polycrystalline sample of TmZn was prepared by induction melting of the high purity Tm and Zn elements in a sealed Ta-tube. Firstly, high purity Tm and Zn with stoichiometric amounts were weighted and arc-welded in a Ta-tube under an argon pressure of ca. 75 kPa. Then the Ta-tube was placed in a water-cooled sample chamber of an induction furnace and heated up to 1250 K for five minutes, following by two hours annealing at 950 K. The sample was proved to be single phase by X-ray powder diffraction and Energy Dispersive X-ray Spectroscopy. The magnetization measurements under various hydrostatic pressures with DC magnetic fields up to 5 T were performed with a commercial superconducting quantum interference device (SQUID) magnetometer by Quantum Design (MPMS-5S) from 2 to 32 K. The sample was compressed in a homemade micro-CuBe pressure cell which was filled with the mixture of Florinerts 70 and 77 as the pressure transmitting medium. The hydrostatic pressure inside the cell was determined by the superconducting transition temperature of Sn.

## Additional Information

**How to cite this article:** Li, L. *et al*. Hydrostatic pressure effect on magnetic phase transition and magnetocaloric effect of metamagnetic TmZn compound. *Sci. Rep.* **7**, 42908; doi: 10.1038/srep42908 (2017).

**Publisher's note:** Springer Nature remains neutral with regard to jurisdictional claims in published maps and institutional affiliations.
